# The detection and prediction of surgical site infections using multi-modal sensors and machine learning: Results in an animal model

**DOI:** 10.3389/fmedt.2023.1111859

**Published:** 2023-04-17

**Authors:** Charmayne Mary Lee Hughes, Andrew Jeffers, Arun Sethuraman, Michael Klum, Milly Tan, Valerie Tan

**Affiliations:** ^1^ Health Equity Institute NeuroTech Laboratory, San Francisco State University, San Francisco, CA, United States; ^2^ Crely Healthcare Pte. Limited, Singapore, Singapore

**Keywords:** surgical site infection, multi-modal sensors, non-invasive sensors, machine learning, wound healing, superficial incisional infection, animal model

## Abstract

**Introduction:**

Surgical Site Infection (SSI) is a common healthcare-associated infection that imposes a considerable clinical and economic burden on healthcare systems. Advances in wearable sensors and digital technologies have unlocked the potential for the early detection and diagnosis of SSI, which can help reduce this healthcare burden and lower SSI-associated mortality rates.

**Methods:**

In this study, we evaluated the ability of a multi-modal bio-signal system to predict current and developing superficial incisional infection in a porcine model infected with Methicillin Susceptible Staphylococcus Aureus (MSSA) using a bagged, stacked, and balanced ensemble logistic regression machine learning model.

**Results:**

Results demonstrated that the expression levels of individual biomarkers (i.e., peri-wound tissue oxygen saturation, temperature, and bioimpedance) differed between non-infected and infected wounds across the study period, with cross-correlation analysis indicating that a change in bio-signal expression occurred 24 to 31 hours before this change was reflected by clinical wound scoring methods employed by trained veterinarians. Moreover, the multi-modal ensemble model indicated acceptable discriminability to detect the presence of a current superficial incisional SSI (AUC = 0.77), to predict an SSI 24 hours in advance of veterinarian-based SSI diagnosis (AUC = 0.80), and to predict an SSI 48 hours in advance of veterinarian-based SSI diagnosis (AUC = 0.74).

**Discussion:**

In sum, the results of the current study indicate that non-invasive multi-modal sensor and signal analysis systems have the potential to detect and predict superficial incisional SSIs in porcine subjects under experimental conditions.

## 1. Introduction

Despite the advances in peri- and intraoperative prevention measures, surgical site infections (SSIs) remain one of the most common adverse events that occur in patients undergoing in-patient or outpatient surgical procedures. SSIs can be classified as either superficial-incisional (involving the skin or subcutaneous tissue layers of the incision), deep-incisional (involving muscle or connective tissue layers of the incision), and organs/spaces deep to the incision that were opened or manipulated during surgery (Table 1) (1). Of the three, superficial incisional SSI are more common than deep incisional and organ/space SSI (2, 3), and account for more than half of all SSIs for all surgery categories (e.g., cardio-thoracic, gynecologic, orthopedics, transplant) (2).

**Table 1:** Surgical site infection classification according to the centers for disease control and prevention and national healthcare safety network [2022].

Type	Definition
Superficial Incisional	Date of event occurs within 30 days following the NHSN operative procedure (where day 1 = the procedure date) *and* involves only skin and subcutaneous tissue of the incision *and* patient has at least one of the following: a) Purulent drainage from the superficial incision. b) Organism(s) identified from an aseptically-obtained specimen from the superficial incision or subcutaneous tissue by a culture or nonculture based microbiologic testing method which is performed for purposes of clinical diagnosis or treatment [for example, not Active Surveillance Culture/Testing (ASC/AST)]. c) A superficial incision that is deliberately opened by a physician or physician designee and culture or non-culture based testing of the superficial incision or subcutaneous tissue is not performed *and* patient has at least one of the following signs or symptoms: localized pain or tenderness; localized swelling; erythema; or heat. d) Diagnosis of a superficial incisional SSI by a physician or physician designee
Deep Incisional	Date of event occurs within 30 or 90 days following the NHSN operative procedure (where day 1 = the procedure date) *and* involves deep soft tissues of the incision (for example, fascial and muscle layers) *and* patient has at least one of the following: a) Purulent drainage from the deep incision. b) A deep incision that is deliberately opened or aspirated by a physician or physician designee or spontaneously dehisces *and* organism(s) identified from the deep soft tissues of the incision by a culture or non-culture based microbiologic testing method which is performed for purposes of clinical diagnosis or treatment or culture or nonculture based microbiologic testing method is not performed *and* patient has at least one of the following signs or symptoms: fever (>38°C); localized pain or tenderness. c) An abscess or other evidence of infection involving the deep incision detected on gross anatomical exam, histopathologic exam, or imaging test.
Organ/Space	Date of event occurs within 30 or 90 days following the NHSN operative procedure (where day 1 = the procedure date) *and* involves any part of the body deeper than the fascial/muscle layers that is opened or manipulated during the operative procedure *and* patient has at least one of the following: a) Purulent drainage from a drain placed into the organ/space. b) Organism(s) identified from fluid or tissue in the organ/space by a culture or non-culture based microbiologic testing method which is performed for purposes of clinical diagnosis or treatment. c) An abscess or other evidence of infection involving the organ/space detected on gross anatomical exam or histopathologic exam, or imaging test evidence definitive or equivocal for infection.

Physician may be interpreted to mean surgeon, infectious disease physician, emergency physician. Physician's designee may be interpreted to mean a nurse practitioner or physicians’ assistant.

Regardless of SSI classification, SSIs place a significant burden on both the patient and health system. In the US alone, SSI extends the hospital length of stay by 9.7 days (4) and is associated with a 2- to 11- fold increase in the risk of mortality (5, 6). SSI complications translate into $10 billion in additional healthcare costs (5, 7), with an average of $25,000 excessive cost per case (5, 7).

Focusing specifically on antibiotics to treat SSI, Jenney et al. (8) reported that the wholesale cost of antibiotics to treat SSI after coronary artery bypass surgeries (CABGs) was AUD $391 (AUD $727.43 inflated to 2022 prices). The cost of antibiotic treatment for patients undergoing vascular surgery who developed an SSI was £3,776 (£4,280.25 inflated to 2023 prices) (9). Antibiotics after major head and neck surgery due to MRSA infection was £260 (£429.54 inflated to 2023 prices) on first admission and £1,700 (£2,808.56 inflated to 2023 prices) on each re-admission (10). As such, SSI imposes an enormous clinical and economic burden on both patients and healthcare systems.

Over the past two decades, healthcare systems have implemented strategies that minimize the length of hospital stay and move inpatient surgical procedures to the outpatient setting. Consequently, a growing proportion of SSIs are detected only after discharge (11). Indeed, of the 500,000 SSIs that occur in the U.S. annually, an estimated 69% of SSIs occur after hospital discharge (11). At present, there is no international scientific consensus about the optimal method for post-discharge SSI surveillance (12). Common methods for identifying surgical wound infection after hospital discharge include direct observation by healthcare professional, telephone interview, patient questionnaire, and outpatient clinic follow-up (13). Because post-discharge surveillance remains unstructured, SSIs are often overlooked (14, 15), so there may be a significant time delay before the physician is able to detect the infection. This is unfortunate, given that a 45-hour delay in detection and treatment of an SSI increases the odds of infection related deaths by 3.8 times (6, 16, 17).

With increasing demand for healthcare, SSIs need to be predicted and diagnosed early so that timely and effective treatment (e.g., antibiotics) can be implemented to accelerate the recovery of patients (4, 18–20). Fortunately, normal surgical wound healing processes occur in a defined and organized fashion, requiring oxygen to promote constructive healing and fight off any contaminants or organisms (21). The development and/or presence of infection leads to directly observable changes (e.g., swelling and erythema) (22) that directly reflect changes in tissue physiology (e.g., abnormal tissue oxygenation, altered acid base, and temperature changes) (23). Such changes can be measured using sensor-enabled technologies with sophisticated signal processing techniques (24–28). For example, Govinda et al. [2010] reported that upper arm subcutaneous oxygen partial pressure recorded using near-infrared spectroscopy 75 min after colorectal surgery predicted SSI with a sensitivity of 71% and specificity of 60%. More recently, Mostafalu et al. [2018] developed a wound dressing capable of continuously measuring wound pH and temperature connected to a wireless electronic module, with *in vitro* bacterial testing indicating that the system could accurately and reliably record wound potential of hydrogen [pH] and temperature.

Taken together, these contribute to the growing evidence that the status of wound healing can be objectively quantified using digital devices. The current study builds on this research by developing a multi-modal bio-signal system equipped with clinical-grade sensors capable of continuously monitoring peri-wound tissue oxygen saturation (StO2), temperature, and bioimpedance (BioZ). The ability of the developed system to predict developing infection in a porcine model was evaluated using a bagged, stacked, and balanced ensemble logistic regression machine-learning model. These results are the first step towards determining the efficacy of a multi-modal sensor system to collect digital biomarkers continuously from the surgical site to monitor the healing status of the wound, with the goal of eventually developing a machine-learning based early warning system to detect and predict SSIs that could become the standard of care for remotely monitoring patients' post-surgery. The aim of the present study was to evaluate the ability of a multi-modal bio-signal acquisition system to predict current and developing superficial incisional infection in a porcine model infected with Methicillin Susceptible Staphylococcus Aureus (methicillinsusceptible S. aureus, MSSA).

## 2. Materials and methods

### 2.1. Sensor apparatus

**Figure 1:**
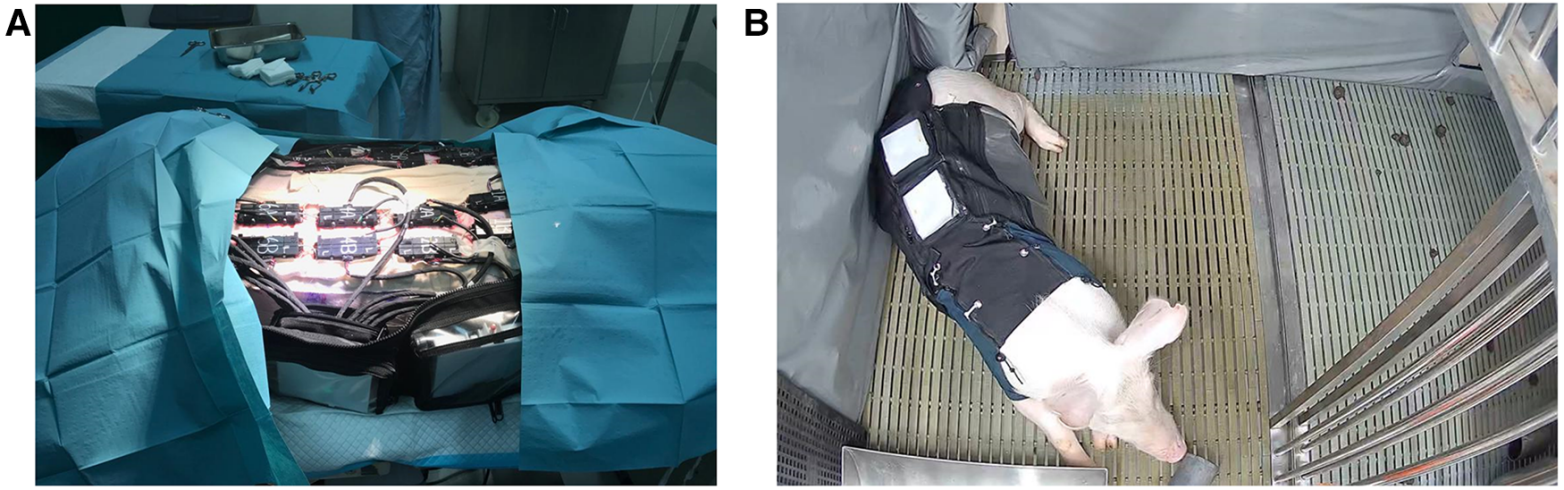
(**A**) CrelySENSE sensors placed on a porcine subject. (**B**) Porcine subject wearing the customized jacket in their individual pen.

Wound healing data was collected using a multi-modal bio-signal system (Figure 1, Crely Healthcare Ltd. Pte., Singapore) that continuously measures peri-wound site temperature, tissue oxygen saturation (StO2), and bioimpedance (BioZ). The multi-modal biosignal system (CrelySENSE) consists of a printed circuit board (PCB) that incorporates a custom-built near-infrared spectroscopy (NIRS) subsystem, a digital temperature sensor (TMP117, Texas Instruments), a bioimpedance system (AD5941) capable of generating high frequency signals up to 200 kHz, and an inertial measurement unit module containing a 3D accelerometer and 3D gyroscope (LSM6DS33, STMicroelectronics). The NIRS subsystem uses an AFE4420 analog front-end to collect optical biosensing information, and features two integrated light emitting diodes (LEDs, SMT730D/850D, Marubeni, Tokyo, Japan) that emit red of 730 nm and infra-red light at 850 nm, and a silicon-photodiode with a large active area (7.5 mm^2^) that was used as photodetector (VEMD5060X01, Vishay Intertechnology Inc., USA). The LEDs and photodiode were soldered on the PCB at a source-detector separation distance of 50 mm. Sensor data was continuously acquired *via* regular burst-mode sampling (50 Hz), whereby data was captured in one-minute bursts over a 10-minute interval. The electronic components were encased in silicon molding, with dimensions of 10 cm × 6 cm × 3 cm and a total weight of 121 g.

The CrelySENSE sensors interfaced with an eight-channel bio-signal acquisition system (CrelyPRO) that featured onboard data storage (Figure 1B). The CrelyPRO bio-signal acquisition system utilizes the Feather nRF52840 single chip solution by Adafruit, PCF8523 CMOS1 Real-Time Clock (RTC, Adafruit), and was powered by a 3.7v Lithium Polymer battery. The material cost of the system, which includes the CrelyPRO bio-signal acquisition system and 28 CrelySENSE sensors [2 sensors per incision], was USD $20,000.

### 2.2. Animal model selection and wound

The anatomical, physiological, and immune system of the pig closely parallel that of humans (29). Porcine and human skin share numerous similarities, with relatively thick epidermis, distinct dermal papillae, and dense elastic fibers in the dermis observed in both species. Similar to humans, pigs have a sparse hair coat and firmly attached skin that adheres to underlying soft tissue structures, and also demonstrate similar epidermal turnover time and keratinous protein characteristics. Porcine skin is dissimilar to human skin in that the dermis of the pig has poorer vascularization, has smaller holocrine sebaceous glands, and lacks eccrine sweat glands. A comparative review of 180 empirical wound healing studies (30) reported that porcine models are concordant with human studies 78% of the time. In contrast, the concordance between wound healing in humans and small mammal (e.g., rabbit, guinea pig, mouse, rat) and *in vitro* studies is much lower (i.e., 53% and 57%, respectively). In sum, the similarities between pig and human skin and wound healing make the pig an appropriate and accurate model to examine biomarkers that characterize wound healing and superficial incisional infection from MSSA.

### 2.3. Animal preparation and surgical protocol

Two skeletally-mature pigs (*Sus scorfa domestica*) (weighing between 80 and 90 kg) were used in the current experiment, and were treated in accordance with animal procedures and applicable animal welfare regulations outlined by the SingHealth Institutional Animal Care and Use Committee (IACUC) guidelines (Ref 2020/SHS/1588). Prior to the start of the experiment (Day -9), animals were fitted with a customized jacket that enabled adequate access to the testing sites, while also ensuring that the sensors and surgical sites were protected from perturbations (e.g., scratching or bumping). Each animal was housed in an individual pen for a seven-day acclimatization period, during which trained veterinary personnel attended to their physiological needs daily and regularly monitored the fit of the jacket to ensure that their mobility and flexibility were not heavily restricted.

**Figure 2:**
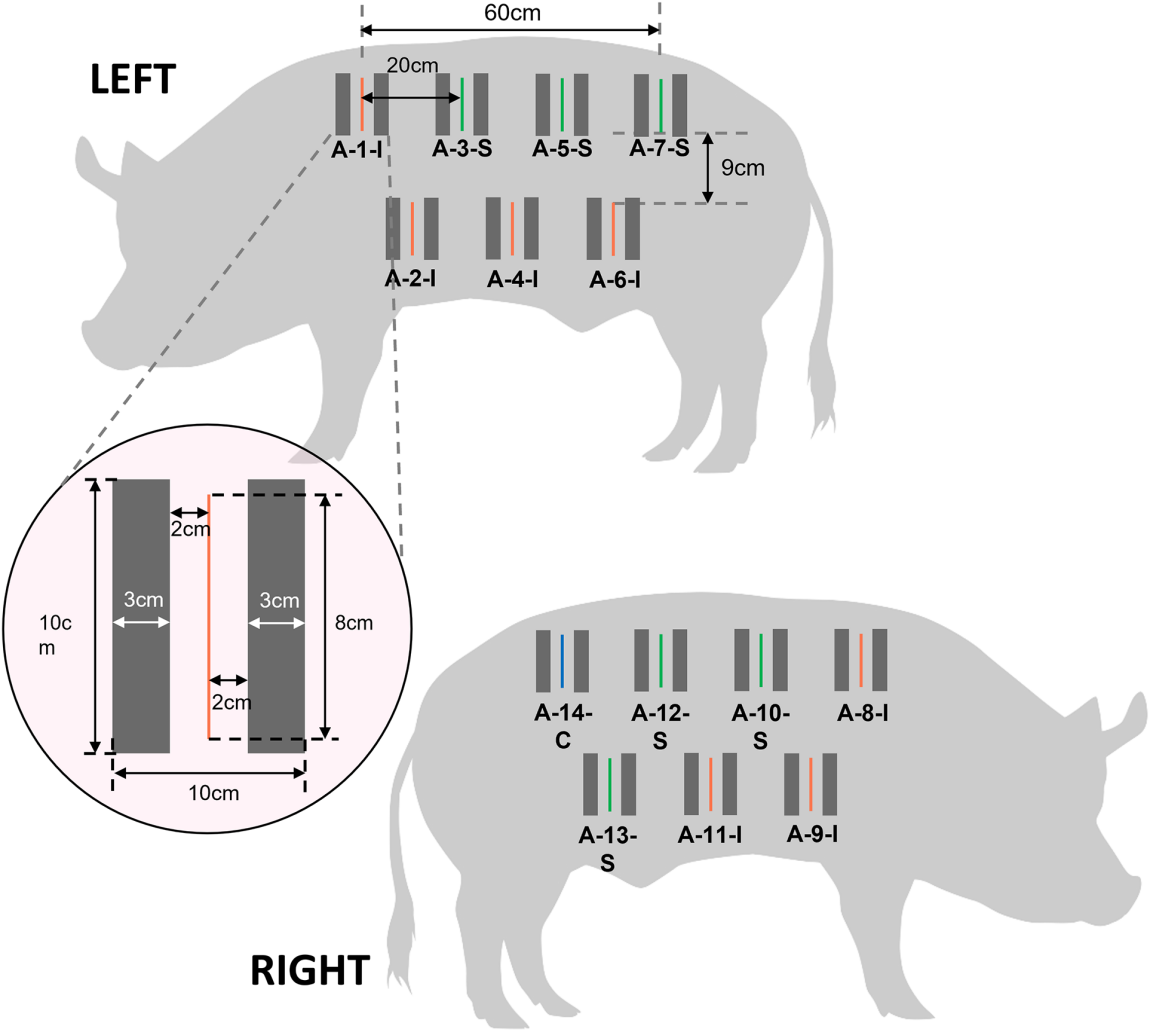
Schematic illustrating the layout of incisions and placement of CrelySENSE sensors. Inoculated wounds are indicated in red, sham wounds in green, and the control site in blue.

Three days prior to wound creation procedure (Day -3), the pigs were fasted overnight and sedated with 15–20 mg Ketamine and 2–5 mg/kg Diazepam. As the NIRS subsystem of the CrelySENSE sensors must remain incident with the skin, the lateral region of the pigs were shaved and scrubbed with a 0.05% Chlorhexidine solution. Subsequently, a CrelySENSE sensor was placed 2 cm on either side of 14 testing sites (Figure 2) and secured to the skin using 3-0 silk sutures. Vital parameters (Heart rate [HR], respiratory rate [RR], oxygen saturation [Sp02], and body temperature) were recorded using a pulse oximeter and a manual thermometer. Buprenorphine 0.01–0.05 mg/kg was administered intramuscularly, with dosage managed by the veterinarian depending on the animal's vital parameters and observed clinical signs of pain or distress.

On the day of surgery (Day 0) the pigs were fasted overnight. Thirty minutes before the anesthetic induction, Buprenorphine 0.01–0.05 mg/kg was administered intramuscularly (IM). Ketamine 15–20 mg/kg, Diazepam 2–5 mg/kg and Atropine Sulfate 0.05 mg/kg (to prevent bradycardia) was administered IM while the animal was in the pen by trained veterinary personnel. When the pigs are in plane 2 stage of anesthesia, they were induced with 4%–5% Isoflurane *via* inhalation for 3–5 min and intubated by the veterinarian using an appropriate sized endotracheal tube. A 21–23 G intravenous catheter was placed in the ear vein for infusion of 0.09 Sodium Chloride (Na Cl) while the animal was undergoing surgery. The animal was sent to the operating room and maintained under 2%–3% Isoflurane. The animal was positioned in lateral recumbency, and the fore/hind legs were secured with cloth tie. Patient monitor sensors were attached to the animal to monitor the vital parameters (HR, RR, Sp02, body temperature, blood pressure (BP), and end-tidal carbon dioxide (ETCO2). In alignment with IACUC policies of animal care and use, the dorsum of each pig was shaved and aseptically prepared for surgery using 70% Ethanol and 0.05% Chlorhexidine solution. Portions of the jacket were removed to render the aforementioned 14 sites easily accessible. The testing sites were draped using sterile drape. Full thickness, 8 cm long incisions transacting the epidermis, the dermis, and the subcutaneous layer without entering the fascia of the musculature (cutaneous trunci/ latissimus dorsi) were performed using a size 21 scalpel blade at 13 of the 14 sites, leaving one site as a control. Seven incisions were inoculated with 100 microliters of 10^9 ^CFU/ml Methicillin Susceptible Staphylococcus Aureus (ATCC 29213) under aseptic, surgical conditions, with the inoculum kept out of contact with the surrounding skin.

Each pig was subjected to the continuous collection of digital biomarkers (wound temperature, tissue oxygenation level, and bioimpedance) by Crely sensors from Day -3 through Day 7. Every day, each pig had its vitals (heart rate, respiratory rate, blood oxygen saturation and core temperature) measured using a handheld pulse oximeter and manual thermometer. Subsequently, each wound was monitored for clinical signs of infection (redness, drainage, etc) through a manual wound assessment performed by the facility veterinarian. All observations were recorded in physical examination sheet and filed in the project folder. Skin biopsy was performed daily on Days 2–7. Biopsies were performed while the animal was under light anesthesia, except on Day 7 when the pig was under general anesthesia. During the biopsies, the jacket remained on the pig as it contained openings that render the sites easily accessible by the veterinarian performing the procedure. A size 21 surgical blade and a pair of surgical scissors were used to extract a 10 mm × 3 mm × 10 mm piece of tissue from two wound sites per day. The incisional biopsy transacted the epidermis, dermis and subcutaneous layers, with the original incision lying to either the left or right edge of the extracted tissue.

On Day 7, the pigs were euthanized with an IV injection of 60–80 mg/kg Pentobarbital Sodium. After tissue harvest, the carcass was disposed as per standard operating procedures.

### 2.4. Clinical wound scoring

Consistent with prior research in animal populations (31, 32), each wound was monitored by two blinded veterinarians for clinical signs of infection using the United States Centers for Disease Control and Prevention (CDC) criteria (33). A wound score was assigned daily based on the scoring criteria (Table 2).

**Table 2:** Wound scoring system.

Wound characteristic	Scoring system
Dehiscence	0 = No dehiscence
	1 = Mild dehiscence
	2 = Moderate dehiscence
	3 = Severe dehiscence
Discharge	0 = No discharge
	1 = Serous discharge
	2 = Seropurulent discharge
	3 = Purulent discharge
Redness	0 = No redness
	1 = Redness at cruciate suture
	2 = Redness around entire incision
	3 = Redness beyond the borders of the incision
Swelling	0 = No swelling
	1 = Mild swelling
	2 = Moderate swelling
	3 = Severe swelling

### 2.5. Multi-modal ensemble machine learning model

Multi-modal, bagged, stacked, and balanced ensemble logistic regression machine learning models were developed to predict the presence of a current superficial incisional SSI, an SSI 24 h in advance of veterinarian-based SSI diagnosis, and an SSI 48 h in advance of veterinarian-based SSI diagnosis. In the first step, the signals were filtered and motion-derived artifacts were removed from the raw signal, after which 90 features (e.g., slope and percentiles) were extracted from the processed data (5,860 observations from 35 wound sites) using a moving window approach. The minority classes were up-sampled to account for class imbalance in the data, and the models were trained using a bootstrap aggregation ensemble technique. Throughout the training with entire data sets, we measured the performance, specificity, and accuracy of each test set, and reported the averaged performance. Then, the probabilities and confidence of intervals were obtained *via* fusion of predictions for multiple classifiers. The model was trained and validated using Leave-1-Out Cross-Validation (34).

### 2.6. Statistical analysis

The first step of the analysis focused on exploring differences in the expression levels of the individual biomarkers (i.e., StO2, temperature, and BioZ) due to the presence of superficial incisional infection. These were determined by conducting two-tailed unpaired Student's t-tests between infected and non-infected surgical sites, separately for each time point. Second, cross-correlations were used to compare discrete time points between the veterinarian-based and bio-signal-based SSI diagnosis for each bio-signal of interest (i.e., temperature, StO2, BioZ). In doing so, it enabled the determination of whether, and at what time lag, the strongest relationship exists between the SSI measures (35). Cross-correlation coefficients with values < 0.20 were classified as very weak, values between 0.20–0.40 as weak, values between 0.40–0.70 as moderate, values between 0.70–0.90 as strong, and values > 0.90 as very strong (36). Statistical analyses were performed using the MATLAB software package version R2021A (The MathWorks, Natick, Massachusetts), with a *p*-value < 0.05 indicating statistical significance.

Third, the ability of the ML model to detect the presence of superficial incisional SSI, predict superficial incisional SSI 24 h in advance of veterinarian-based SSI diagnosis, and predict superficial incisional SSI 48 h in advance of veterinarian-based SSI diagnosis was determined. Given that accuracy is likely to be overestimated in unbalanced data, model performance was evaluated using the following metrics: area under the ROC curve (AUC), accuracy, specificity, and sensitivity (36). The overall diagnostic accuracy of the model was determined using Hosmer & Lemeshow's (38) empirical classifications, in which an AUC < 0.5 indicates no discrimination, 0.7–0.8 indicates acceptable discriminability, 0.8–0.9 indicates excellent discriminability, and > 0.9 indicates outstanding discriminability. Pairwise comparison of AUCs was performed using DeLong's test implemented in the R package pROC (R version 4.1.1, The R Foundation) (39). The *P* values of multiple comparison of AUCs were adjusted by Bonferroni correction and tests with *p* < 0.017 were interpreted as a significant difference.

## 3. Results

A total of 14 incisions were made on each pig: seven were inoculated with MSSA, six served as sham sites, and one served as a control site. For pig #1, 5/7 (71.4%) inoculated sites were infected, 1/6 sham wound sites were infected (14.3%), and 0/1 (0%) control site were infected. For pig #2, 6/7 (85.7%) inoculated sites were infected, 0/6 sham wound sites were infected (14.3%), and 0/0 (0%) control sites was infected.

A total of 28 Hematoxylin and Eosin (H&E) stained skin tissue samples were scored by a certified Research Veterinary Pathologist for histopathological changes such as inflammation, granulation tissue, collagen deposition, epithelization and neovascularization based on the score pattern described by Barington et al. (40). The infiltration of polymorphonuclear cells (PMNCs) persist in inoculated biopsy skin samples from days one though six, decreasing in distribution until day five, with a sudden increase in presence on days three and six. The infiltration of PMNCs in sham sites is decreased compared to inoculation sites on various days of surgical removal of biopsy. The presence of mononuclear cells in inoculation sites increased from day 1–5, and comparatively declined in distribution in sham sites. Samples taken from the control site failed to show pathological changes.

**Figure 3:**
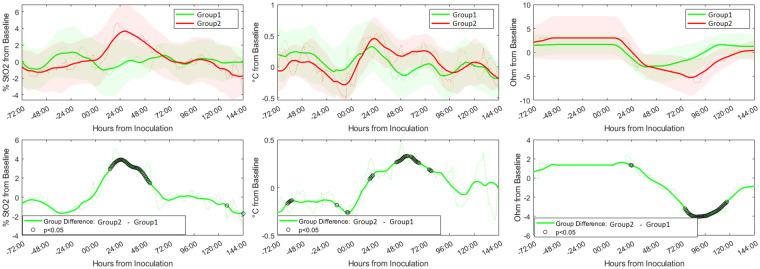
Expression of digital biomarkers in the first six days after inoculation with methicillin susceptible Staphylococcus Aureus (MSSA). Graphs show the relative trends of peri-wound tissue oxygen saturation (**left** panel), temperature (**middle** panel), and bioimpedance (**right** panel) from the sham wounds (green) and inoculated wounds (red). The difference in expression levels of the individual biomarkers with time showed significant differences between the two groups [marked with black rings (i.e., ○)] at certain time points (**bottom** panel).

Figure 3 (top panel) illustrates time-related differences in peri-wound tissue oxygen saturation (% StO2), wound temperature (temperature from baseline), and bioimpedance (BioZ from baseline) for non-infected (green) vs. superficial incisional infected wounds (red) relative to the sham wounds. Statistical differences between wounds are indicated with black rings (i.e., ○). The percentage of tissue oxygenation (% StO2) for infected wounds followed a bell curve pattern under 72 h post-inoculation peaking at the 25th hour. This differed from non-infected wounds that were relatively stable across the study period. Statistical analysis indicated that differences in percent StO2 between the infected and non-infected surgical sites reached statistical significance at 12 h post-inoculation until 50 h post-inoculation (Figure 3 bottom left panel, all *p*'s < 0.05). Wound temperature (°C from baseline) between non-infected and infected wounds demonstrated similar trends over the study period, but with markedly different values that started 18 h post-inoculation and lasted until 96 h post-inoculation (Figure 3 bottom middle panel). However, statistical analysis indicated that the differences in wound temperature between the infected and non-infected surgical sites reached statistical significance only during the time period between 24 until 72 h post-inoculation (all *p*'s < 0.05). Wound bioimpedance (Ohm from baseline) between non-infected and infected wounds demonstrated similar trends over the study period, but with markedly different values that started 48 h post-inoculation and lasted until 120 h post-inoculation (Figure 3 bottom right panel). However, statistical analysis indicated that the differences in wound bioimpedance between the infected and non-infected surgical sites reached statistical significance only during the time period between 72 until 120 h post-inoculation (all *p*'s < 0.05).

**Figure 4:**
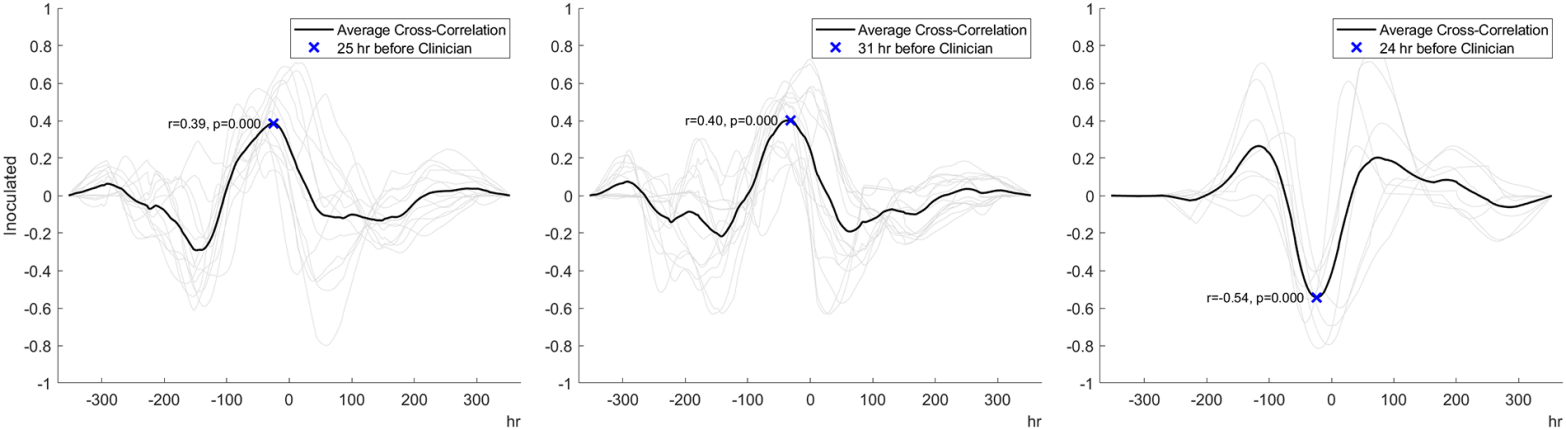
Cross-correlation analysis data indicating that peri-wound tissue oxygenation (**left** panel), temperature (**middle** panel), and bioimpedance (**right** panel) predict the development of infection 24 to 31 h before veterinarian-based SSI diagnosis.

For superficial incisional infected wounds, cross-correlational analysis indicated a change in the three bio-signals that occurred at least 24 h before a change in veterinarian-based SSI diagnosis (Figure 4). The percentage of tissue oxygenation (% StO2) for infected wounds exhibited a significant upward trend 25 h in advance of a clinician's diagnosis of wound infection (*r* = 0.39, *p* < 0.001). Similarly, the peri-wound temperature exhibited a moderately upward trend at 31 h before a diagnosis of infection (*r *= 0.40, *p* < 0.001). A moderately strong downward trend in the BioZ value of the tissue was observed 24 h prior to the clinical diagnosis of wound infection (*r* = 0.54, *p* < 0.001).

Table 3 presents the ML model's ability to predict current and future superficial incisional SSI. The ability of the model to detect a current SSI achieved an AUC of 0.77, accuracy of 64%, sensitivity of 82%, and specificity of 59%. In comparison, the predictive performance of the model was higher when predicting SSI 24 h in advance of veterinarian-based SSI diagnosis, with an AUC of 0.80, accuracy of 78%, sensitivity of 81%, and specificity of 76%. The algorithm exhibited lower performance for predicting SSI 48 h in advance of veterinarian-based SSI diagnosis (AUC = 0.74, accuracy = 69%, sensitivity = 77%, specificity = 65%). By pairing and comparing the AUC using DeLong's test, we found that there was no statistical difference between predicting current SSI and 24 h in advance of veterinarian-based SSI diagnosis (*p* = 0.071), between current and 48 h in advance of veterinarian-based SSI diagnosis (*p* = 0.0790), while the difference between predicting SSI 24 h in advance of veterinarian-based diagnosis and predicting SSI 48 h in advance of veterinarian-based SSI diagnosis was statistically significant (*p* < 0.001).

**Table 3:** Machine learning model performance in detecting the presence of a current SSI, SSI 24 h in advance of veterinarian-based SSI diagnosis, and SSI 48 h in advance of veterinarian-based SSI diagnosis.

	AUC	Accuracy	Sensitivity	Specificity
Current SSI	0.77	64%	82%	59%
24 Hours	0.80	78%	81%	76%
48 h	0.74	69%	77%	65%

## 4. Discussion

The aim of the present study was to evaluate the ability of a multi-modal bio-signal acquisition system to predict current and developing superficial incisional infection in a porcine model infected with Methicillin Susceptible Staphylococcus Aureus. The predictions of the system were evaluated using a bagged, stacked, and balanced ensemble logistic regression machine learning model.

The acquired bio-signals enabled the characterization of wound healing and superficial incisional infection from MSSA across the study period. At non-infected wound sites, StO2 was relatively stable from the point of inoculation to the end of the study period, temperature increased from the point of inoculation until the 24th hour then decreased gradually, and bioimpedance decreased from the point of inoculation to the 80th hour, then increased to the end of the study period. In contrast, StO2 of infected wounds increased from the point of inoculation to the 25th hour then decreased to the 80th hour. Temperature and bioimpedance of infected wounds showed similar trends to the non-infected wounds, but with higher peri-wound temperatures from the 24th hour through the 96th hour, and lower bioimpedance values from the 48th hour through the 120th hour. When comparing the values of the individual biomarkers to clinical wound scores, the results of the present study demonstrate that digital signals can indicate the presence of an MSSA infection 24 to 31 h before standard methods. The ability to detect changes in tissue physiology associated with SSI is of critical importance to healthcare providers, as it would help enable the timely and effective treatment (e.g., antibiotics) to accelerate the recovery of patients (4, 20), that would ultimately reduce healthcare costs, SSI-associated complications and mortality rates.

Interestingly, there were statistically significant differences between the inoculated and sham wound sites 60–55, 10, and 2–1 h prior to the incision and introduction of MSSA. Because the distribution of inoculated and sham wounds across the trunk of the animals, it is unlikely to be due to local physiological effects. Rather, because the *p*-values during these time periods range from *p *= 0.041 to 0.047 we believe that the statistical significance at these time periods are spurious in nature, and be non-significant with more data. We minimized the number of animals involved in the current study, because the experimental research methodology was invasive by its very nature which resulted in potential undue distress and harm on the animals. As the research moves into post-surgical human populations, the non-invasive observational research methodologies (i.e., monitoring post-surgical patients) will allow us to evaluate the system in a greater number of wound sites. If our assumption regarding statistical spuriousness is correct, then we expect that pre-surgical temperature will be similar within an anatomical region.

Congruent with prior studies that have developed machine-learning powered SSI models using medical data (41) or wound imaging (42), the multi-modal ensemble model developed in this study demonstrated promising accuracy and sensitivity to detect a current infection, yielding an AUC value of 0.77. While model performance to detect SSI 24 h and 48 h in advance of veterinarian-based SSI diagnosis (AUC's = 0.80 and 0.74, respectively) also demonstrated acceptable discriminability, DeLong's tests indicated that both the current infection and the 24 h in advance models performed equally well, whereas the model predicting SSI 48 h in advance of veterinarian-based SSI diagnosis performed significantly worse. Based on the data, it can be put forth that the measured bio-signals are best at providing information regarding pathophysiological processes before overt clinical signs are apparent. This conjecture is supported by results of the cross-correlation analysis indicating that peri-wound bioimpedance and tissue oxygenation of inoculated wounds peaked 25 and 24 h before veterinarian-based SSI diagnosis. Taken together, the current data indicate that superficial incisional wounds infected with MSSA result in physiological changes to the peri-wound area (e.g., rise in peri-wound temperature due to inflammation, immune responses, and/or tissue metabolism) that can be detected using non-invasive multi-modal sensors.

While the model showed acceptable performance, the diagnostic accuracy of the model could be further improved by increasing the volume of training data and/or handling the problem of imbalanced data by using rule-based methods that can learn high confidence rules for the minority SSI class (43) or by building cost-sensitive classifiers (44). Another approach, which may lead to greater clinical adoption, would be to integrate sociodemographic and clinical information (e.g., hypertension, diabetes mellitus, current or past smoker), peri-operative (e.g., urgency of surgery, duration of surgery, surgical drain placed, antibiotic prophylaxis, intraoperative supplemental oxygen), and post-operative parameters (e.g., serous exudate, purulent exudate, white blood cell count, antibiotics administered) into the model.

There are limitations to the current study that may inform future directions in this line of research. First, the sensors were sutured on the skin of the porcine subjects in order to minimize movement-related measurement errors and signal noise. We have adapted the CrelySENSE sensors for use in clinical trials involving human volunteers so that they attach to the peri-wound site *via* disposable adhesives and are connected to the CrelyPRO using Bluetooth low energy (BLE) protocols. Second, SSI may also lead to changes in bio-signals such as perfusion, pulse rate, respiration rate, and pH. The inclusion of these parameters may improve the detection and prediction of SSIs in animal and human populations. Third, we examined responses after MSSA infection. While the most common organism that causes SSIs (45–47), it is unknown whether SSIs caused by bacteria such as Methicillin-resistant Staphylococcus aureus (MRSA), extended-spectrum *β*-lactamase (ESBL)-producing Enterobacteriaceae [Escherichia coli (E. coli), Klebsiella pneumoniae], multidrug resistant gram-negative bacteria (e.g., Acinetobacter baumannii), and vancomycin-resistant Enterococcus faecium (VRE) would yield similar bio-signal responses. Lastly, the current study focused on the detection and prediction of superficial incisional SSIs that occur in the area of the skin where the incision was made. Whether or not the described bio-signal system can detect infections that occur beneath the incisional area in the muscle and surrounding tissues (deep incisional SSIs) remains to be ascertained. There is some evidence that post-surgical deep incisional infections may exhibit different patterns of expression of wound biomarkers (48, 49). Future studies should thus validate the current system in different types of SSIs and pathogens.

In summary, the results of the current study bear out the accuracy, sensitivity, and specificity with which the non-invasive multi-modal bio-signal system of interest is capable of detecting MSSA infection in porcine subjects. Such systems can provide clinicians with a continuous and real-time understanding of the state of the wound healing process, enabling them to make timely decisions regarding wound care that would substantially reduce post-operative complications, length of stay, and treatment costs.

## Data Availability

The raw data supporting the conclusions of this article will be made available by the authors, without undue reservation.
